# Image Encryption Algorithm Based on a New Two-Dimensional Chaotic System and Rotating Dial Model

**DOI:** 10.3390/e28050530

**Published:** 2026-05-07

**Authors:** Xiaoqiang Zhang, Haoran Hu

**Affiliations:** School of Information and Control Engineering, China University of Mining and Technology, Xuzhou 221116, China

**Keywords:** image security, two-dimensional chaotic system, rotating dial model, dynamic diffusion

## Abstract

With the rapid advancement of information technology, the secure transmission and storage of digital images have garnered increasing attention. To safeguard image information from theft and enhance security during network transmission, a novel image encryption algorithm based on a two-dimensional chaotic system named the two-dimensional sine-cubic modular map (2D-SCMM) and a rotating dial model is proposed. First, the 2D-SCMM is designed, and comprehensive dynamic analyses along with randomness assessments are conducted. Second, in the scrambling phase, a diagonal cyclic-shift transformation is employed to dynamically update the distribution of pixel positions. Third, during the diffusion phase, inspired by the dial phone, the rotating dial model is utilized to achieve dynamic pixel updates. Finally, extensive testing and comparative analyses reveal that image pixels are evenly distributed, the average entropy value for grayscale images is 7.9993, and the correlation coefficients approach 0. Meanwhile, the encryption algorithm is highly secure against various attacks, such as noise attacks and cropping attacks.

## 1. Introduction

With the rapid development of today’s information technology, digital images have become an important medium for transmitting, storing, and expressing information, and are widely used in various fields such as healthcare, education, the military, finance, and social media [1]. However, the extensive use and efficient dissemination of image data also pose significant challenges to information security. With the popularization of the Internet and cloud computing technologies, image data face security threats such as unauthorized access, malicious tampering, and data theft during transmission and storage, which may not only result in personal privacy leakage but also lead to serious economic losses and social security risks [2]. Therefore, the study of effective image encryption algorithms to ensure the security of image data has become an important issue in the field of information security.

Traditional encryption algorithms, such as the Advanced Encryption Standard (AES) and the Data Encryption Standard (DES), have demonstrated remarkable efficacy in encrypting text and data with relatively simple structures. However, when applied to digital images, which are characterized by large data volumes, high redundancy, and spatial correlation, these algorithms encounter significant limitations in both efficiency and security [3]. The unique characteristics of digital images necessitate the development of specialized image encryption algorithms. The high spatial correlation inherent in image data makes them particularly vulnerable to attacks, as attackers can readily exploit statistical analysis or selective attacks to extract image information [4]. Additionally, the high redundancy of images demands more efficient encryption strategies to reduce computational complexity and encryption time [5]. With the exponential growth in the demand for high-definition (HD) and ultra-high-definition (UHD) images, the challenge of enhancing encryption efficiency while maintaining robust security has emerged as a focal point of research [6].

Currently, image encryption schemes employ a variety of techniques, including neural networks [7,8], frequency-domain transforms [9,10], cellular automata [11,12], DNA operations [13,14], and chaos theory [15,16], among others. Among these, chaos theory has become a popular technique in the field of image encryption due to its inherent properties, such as sensitivity to initial conditions, inherent randomness, and unpredictability, which are closely related to the principles of cryptography.

Existing chaotic systems are usually categorized into two types: one-dimensional (1D) [17,18,19] and high-dimensional (HD) [20,21,22,23,24]. HD systems are widely used in image encryption due to their higher dimensionality and more complex nonlinear coupling mechanisms. In recent years, many researchers have developed novel chaotic systems and applied them to image encryption. Dong et al. [25] created a new two-dimensional chaotic map called the two-dimensional beta chaotic map (2D-BCM), which exhibits higher sensitivity and security compared with previous schemes. Chen [26] et al. constructed a two-dimensional logistic-sine-Henon map (2D-LSHM) for image encryption. Compared with existing chaotic systems, the 2D-LSHM exhibits better chaotic performance and is more suitable for image encryption scenarios. Fei et al. [27] proposed a new 2D absolute sine–cosine coupling (2D-ASCC) model. The proposed system has higher complexity and better pseudo-randomness than other 2D chaotic maps. Zhang et al. [28] designed a generalized 2D enhanced cosine coupling model (2D-ECCM), which can utilize most existing 1D maps as seed maps to construct new 2D chaotic systems. Therefore, compared with 1D chaotic systems, 2D chaotic systems exhibit more complex dynamical behavior, making them more suitable for image encryption systems and greatly improving the stochasticity and complexity of the encryption process.

However, some existing chaotic systems suffer from drawbacks; for example, their output trajectories are not evenly distributed throughout the phase space, and the resulting chaotic sequences lack randomness. In addition, their chaotic range is small and discontinuous, and within certain parameter ranges, the system may degenerate into periodic behavior rather than hyperchaotic behavior, thereby losing the advantages of HD systems. To overcome these limitations, increasingly improved chaotic maps have been proposed. Huang et al. [29] constructed a new 2D chaotic system by combining two 1D chaotic maps (the logistic map and the ICMIC map) with two nonlinear bounded functions (mod and sine functions). The system utilizes the nonlinear properties and boundedness of modular operations to improve output complexity. Zhou et al. [30] proposed a two-dimensional cross-coupled modular chaos model (2D-CMCM). The system cross-couples two one-dimensional chaotic systems, enabling more complex dynamical behavior than the original systems while also significantly increasing chaos complexity by constraining the output within a finite range using modular operations. Gao et al. [31] designed a new chaotic system (2DSHM) by coupling the sine map and the Henon map and improved its chaotic performance by constraining the output to (0, 1) using modulo 1 operations. Wu et al. [32] designed a 2D discrete hyperchaotic map using exponential functions and modular operations, where the exponential component significantly improves system randomness and traversal. Therefore, we design a new 2D hyperchaotic system using modular operations and exponential components to overcome the limitations of existing chaotic systems.

In general, the typical framework of image encryption is scrambling—diffusion. Scrambling techniques enable the replacement of original image pixel positions and reduce the correlation between adjacent pixels. Scrambling methods include zigzag transforms, spiral transforms, diagonal transforms, and others. Zhang and colleagues [33] constructed a cyclic hierarchical scrambling method, which enhances algorithmic randomness and effectively reduces correlation between neighboring pixels through a parity-layering strategy. Wang et al. [34] designed a bidirectional diagonal cross-transform with different directions, which overcomes the shortcomings of traditional diagonal transforms and significantly reduces horizontal correlation coefficients in planar images. However, these scrambling methods still have limitations, such as their inability to simultaneously reduce correlations among adjacent, vertical, and diagonal pixels. In this paper, a diagonal cyclic-shift transform is proposed to simultaneously reduce the correlation coefficients in the horizontal, vertical, and diagonal directions.

Diffusion techniques modify pixel values to strengthen resistance against statistical attacks. Common diffusion methods include dissimilarity diffusion, add-and-subtract diffusion, and S-box-based diffusion. Zhu et al. [35] constructed an improved two-dimensional diffusion structure that extends pixel-wise transformation from the original image to the entire ciphertext image. Lai et al. [36] combined dissimilarity and add-and-subtract modular operations, resulting in a substantial increase in security. Xu et al. [37] designed a bit-level mutual diffusion technique for grayscale images, which achieves the desired effect in a single round. Wang et al. [38] designed cross-plane scrambling and diffusion combined with S-box substitution, achieving high security through efficient algorithmic design. Wu et al. [32] constructed a new S-box generation method that innovatively combines two chaotic sequences to construct a dynamic S-box that is fast and flexible. However, in order to balance diffusion efficiency and security, we propose a dial diffusion model. The dial model is inspired by physical encryption devices, and its multilayer state-switching and random perturbation mechanisms are highly compatible with the complex dynamics of chaotic systems. Introducing the dial model into the image encryption design further enhances diffusion through layered perturbation and parameter tuning, thereby improving the security and adaptability of the encryption algorithm. The main contributions of this work are as follows:

(1) A new two-dimensional chaotic system named the two-dimensional sine-cubic modular map (2D-SCMM) is proposed. Compared with existing chaotic maps, the 2D-SCMM offers better usability, a larger parameter space, and more complex chaotic behavior.
(2) A novel diagonal cyclic-shift transformation is proposed. It effectively reduces pixel correlation and significantly enhances the scrambling effect.
(3) Inspired by a rotary dial telephone, a diffusion method based on a rotating dial model is proposed. It enables effective image diffusion and further improves the algorithmic security.
(4) An image encryption algorithm is designed by combining the 2D-SCMM, the diagonal cyclic-shift transformation, and the rotating dial model. Experimental results show that the proposed algorithm achieves high security.

The rest of this paper is organized as follows. Section 2 describes the theoretical principles used in the encryption algorithm. Section 3 details the encryption process. Section 4 discusses the experimental results obtained using the proposed algorithm. Finally, the conclusions are drawn in Section 5.

## 2. Theoretical Principles

### 2.1. Chaotic Systems

(1) Sine map

The sine map [39] is a common chaotic map defined mathematically as follows:

(1) $$x_{n+1}=\gamma \sin(\pi x_{n}),$$

where the control parameter *γ* lies in the range *γ* ∈ [0, 1]. The state variable *x* is defined in the range *x* ∈ [0, 1]. When the control parameter *γ* ∈ [0.87, 1], the sine map exhibits chaotic behavior.

(2) Cubic map

The cubic map [40] is a common chaotic map. Compared with the logistic chaotic map, the cubic chaotic map exhibits better chaotic traversal and is characterized by fast convergence and high accuracy. Its expression is as follows:

(2) $$x_{n+1}=\beta x_{n}(1-{x_{n}}^{2}),$$

where the control parameter *β* ∈ [0, 4] and the state variable *x*_n_ ∈ [−1, 1]. When the chaos parameter *β* = 2.595, the cubic chaotic map exhibits good traversal behavior over the interval [0, 1].

(3) Proposed two-dimensional chaotic system

Based on the study of the sine map and the cubic map, the two-dimensional sine-cubic modular map (2D-SCMM) is proposed in this paper. It exploits the nonlinear properties and boundedness of the modulo operation to enhance the complexity of the system output and allow the system to have a larger parameter range. The definition of 2D-SCMM is shown in Equation (3):

(3) $$\left\{\begin{array}{l} x_{n+1}=\mathrm{mod}(\cos(4\pi^{2}(\mu (y_{n}-{y_{n}}^{3})+\sin(\pi^{2}(1-y_{n})))+e^{\mu (x_{n}+y_{n})}),1)\\ y_{n+1}=\mathrm{mod}(\sin(4\pi^{2}(\mu (x_{n}-{x_{n}}^{3})+\cos(\pi^{2}(1-x_{n})))+e^{\mu (x_{n}+y_{n})}),1), \end{array}\right.$$

where the control parameter *μ* lies in the range *μ* ∈ [0, + ∞].

### 2.2. Chaos Performance Analysis

(1) Bifurcation diagram

**Figure 1:**
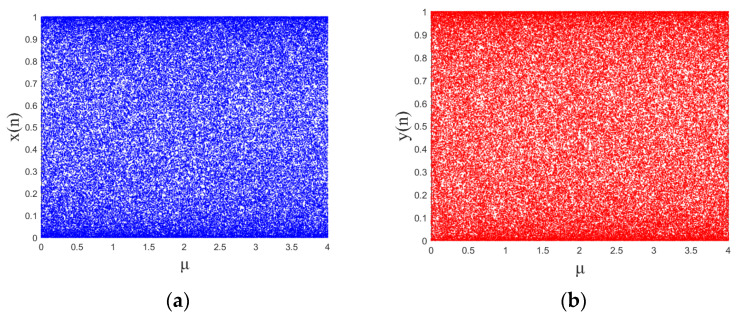
Bifurcation diagram: (**a**) output *X* of 2D-SCMM; (**b**) output *Y* of 2D-SCMM.

The bifurcation diagram [30] shows the state and distribution range of the output sequences in the chaotic map. Given an initial value, the bifurcation map shows how the distribution of values of the pseudo-random sequence changes with variations in the control parameters. Let the initial values of the designed 2D chaotic system be *x*(0) = 0.1 and *y*(0) = 0.1. When the control parameter *μ* ∈ [0, 4], its bifurcation diagram is shown in Figure 1, where Figure 1a shows the evolution of the *x* sequence with variations in the control parameter, and Figure 1b shows the evolution of the *y* sequence with variations in the control parameter. It can be seen that the designed 2D chaotic system is always in a chaotic state, i.e., full-domain chaos, as the control parameters increase.

(2) LE

The Lyapunov exponent (LE) [41] is a key metric for evaluating whether a nonlinear system is chaotic, as it measures the sensitivity of the system to parameters and whether the system enters chaos. Usually, the dimension of a nonlinear system determines the number of LE exponents; i.e., a designed 2D chaotic system has two LEs. The definition of the LE is shown in Equation (4):

(4) $$LE=\underset{n\to \infty }{\lim}\frac{1}{n}\sum_{i=0}^{n-1}\ln\left|f^{\prime }(x_{i})\right|\;,$$

**Figure 2:**
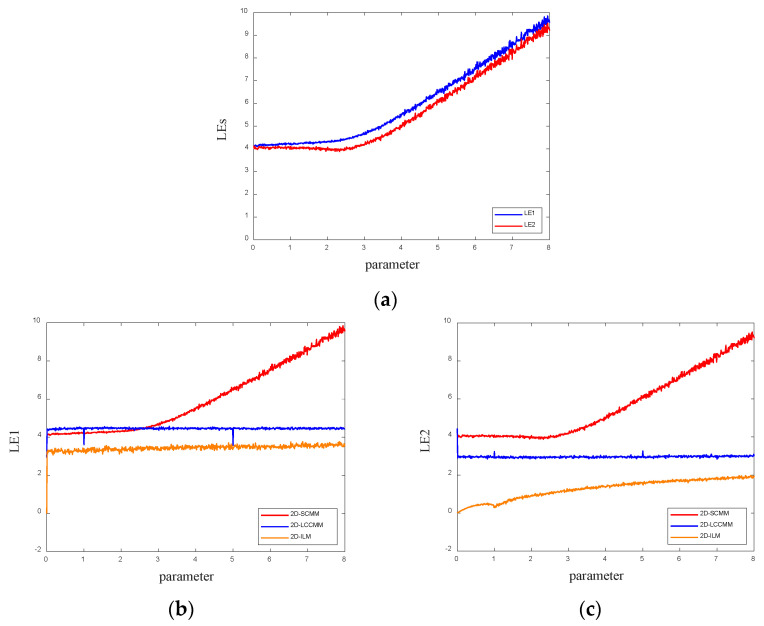
Lyapunov exponent spectra: (**a**) Lyapunov exponent spectra for the 2D-SCMM; (**b**) comparison of LE1; (**c**) comparison of LE2.

When both LEs are greater than 0, it indicates that the system is in a hyperchaotic state. The chaotic system generated by the 2D-SCMM has two LEs. The initial parameter is set as (*x*, *y*) = (0.1, 0.1), and the control parameter is set as *μ* ∈ [0, 8]. Figure 2a visualizes the two LEs of the 2D-SCMM. The proposed map can obtain two positive LEs, which indicates that it can exhibit hyperchaotic behavior. To investigate the performance of the 2D-SCMM, it is compared with other chaotic systems, including 2D-LCCMM [30] and 2D-ILM [29], as shown in Figure 2b,c. It is evident that, overall, the 2D-SCMM exhibits LE values that are generally larger than those of the compared systems across a wide parameter range, particularly for *μ* > 2.5. It is worth noting that in a small parameter interval (*μ* < 2.5), the first LE (LE1) of the 2D-LCCMM is slightly lower than that of the proposed system. However, this does not diminish the overall superior chaotic performance of the 2D-SCMM in the primary parameter range of interest for encryption. This analysis suggests that the 2D-SCMM possesses a broader effective chaotic range and demonstrates more complex hyperchaotic behavior compared to the other maps.

(3) Sensitivity analysis

**Figure 3:**
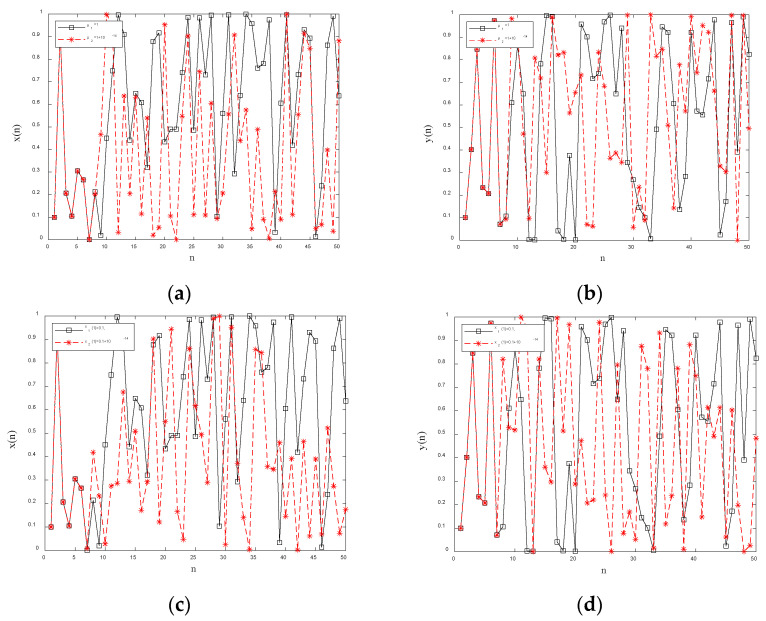
Sensitivity analysis: (**a**) sensitivity of *x* to control parameters; (**b**) sensitivity of *y* to control parameters; (**c**) sensitivity of *x* to initial values; (**d**) sensitivity of *y* to initial values.

Initial condition sensitivity [42] refers to the property that small differences in initial conditions in chaotic systems are exponentially amplified over time, leading to significant deviations in system trajectories. The sensitivity of chaotic systems to initial values determines the sensitivity of encryption algorithms to plaintext. The black solid lines in Figure 3a,b denote the sensitivity of the 2D chaotic system to initial conditions for *μ_1_* = 1, *x*1(0) = 0.1, and *y*1(0) = 0.1 over 50 iterations. Here, n denotes the number of iterations. Keeping the initial values unchanged and changing only the control parameter to *μ_2_* = 1 + 10^−14^, the x-sequence and y-sequence produced by the first 50 iterations of the designed 2D chaotic system are shown as red dashed lines in Figure 3a,b. It can be clearly seen that when *n* = 7, the black solid line and the red dashed line in Figure 3a,b begin to diverge significantly. Therefore, the designed 2D chaotic system can produce almost completely different chaotic sequences when the control parameters are changed slightly, i.e., the designed 2D chaotic system is very sensitive to the control parameters.

The black solid lines in Figure 3c,d represent the x-sequence and y-sequence generated by 50 iterations of the 2D chaotic system under the initial conditions *μ_1_* = 1, *x*1(0) = 0.1, and *y*1(0) = 0.1. Keeping the control parameters unchanged and changing only the initial value to *x*2(0) = 0.1 + 10^−14^, the x-sequence and y-sequence produced by the first 50 iterations of the designed 2D chaotic system are shown as red dashed lines in Figure 3c,d. It can be clearly seen that when *n* = 7, the black solid line and the red dashed line in Figure 3a,b begin to diverge significantly. Therefore, the designed 2D chaotic system is very sensitive to the control parameters.

(4) Sample entropy

**Figure 4:**
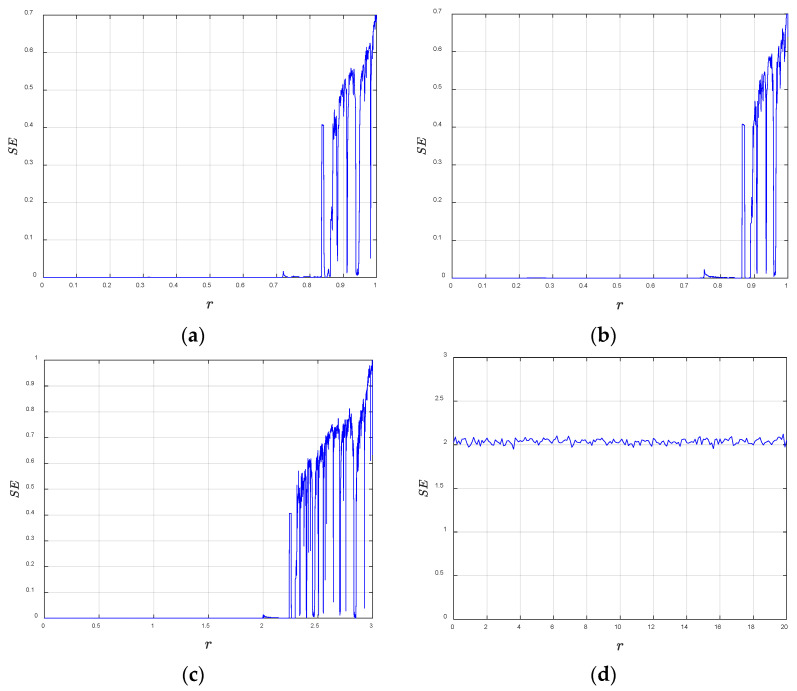
Sample entropy: (**a**) logistic map; (**b**) sine map; (**c**) cubic map; (**d**) 2D-SCMM.

Sample entropy allows the assessment of time-series complexity [43]. The complexity of a nonlinear system increases with the value of the sample entropy. For a time series of size {*x*_1_, *x*_2_, … *x*_N_}, embedding dimension *m,* and similarity tolerance limit *r*, the mathematical expression of sample entropy is as follows:

(5) $$Sample\;entropy(m,r,N)=-\log\frac{A}{B}\;,$$

where *A* and *B* are the numbers of vector pairs satisfying *d*[*X_m_*_+ 1_(*i*), *X_m_*_+ 1_(*j*)] < *r* and *d*[*X_m_*(*i*), *X_m_*(*j*)] < *r*. *d*[*X_m_*(*i*), *X_m_*(*j*)] represents the Chebyshev distance between *X_m_*(*i*) and *X_m_*(*j*). The sample entropy results for the logistic map, sine map, cubic map, and 2D-SCMM are shown in Figure 4. The sample entropy values of 2D-SCMM are higher than those of the other maps, which indicates that the 2D-SCMM has better chaotic performance.

(5) NIST test

The NIST test [44] consists of 15 sub-tests that are primarily used to examine the randomness, uniformity, independence, and other properties of sequences generated by a random number generator, and each test produces a *p*-value. When the *p*-values of all 15 sub-tests in the NIST test are greater than 0.01, the sequence passes the NIST test, i.e., the sequence can be considered random. Let the initial conditions of the designed 2D chaotic system be *μ* = 3, *x*(0) = 0.4, and *y*(0) = 0.6. Two sufficiently long sequences, *x*(*n*) and *y*(*n*), are generated and converted into a binary bit stream for testing. That is, the chaotic sequences *x*(*n*) ∈ [0, 1], *n* = 1,2, …, *N*; *y*(*n*) ∈ [0, 1], *n* = 1,2, …, *N* are converted into binary sequences $x^{\prime }$(*n*) and $y^{\prime }$(*n*), where N is the length of the sequences, and the binary sequences x and y consist of 1 s and 0 s. The conversion method is shown in Equations (6) and (7):

(6) $$x^{\prime }(n)=\mathrm{mod}(floor((x(n)+100)\times 2^{20}),2)\;,$$

(7) $$y^{\prime }(n)=\mathrm{mod}(floor((y(n)+100)\times 2^{20}),2)\;,$$

where *floor*(*x*) rounds the elements of *x* to the nearest integer less than or equal to *x*, and mod(·) stands for the modulo operation. From Table 1, it can be seen that the *p*-values of all sub-tests for *x*(*n*) and *y*(*n*) are greater than 0.01, i.e., the designed 2D chaotic system passes the NIST test. The sequences generated by the 2D-SCMM exhibit a high degree of randomness.

### 2.3. Diagonal Cyclic-Shift Transformation

The traditional diagonal transformation refers mainly to the zigzag transformation, which is realized by traversing the image in a “Z” pattern and then reconstructing the scanned elements into a matrix of the same size as the original image. In this paper, a method combining diagonal transformation and cyclic shift is proposed for scrambling. First, an *m* × *n* image is circularly shifted along the anti-diagonal direction for each anti-diagonal to complete the first scrambling stage. Second, circular shifts are performed along the main diagonal direction for each main diagonal to complete the second scrambling stage, and the number of circular shifts is controlled by chaos.

**Figure 5:**
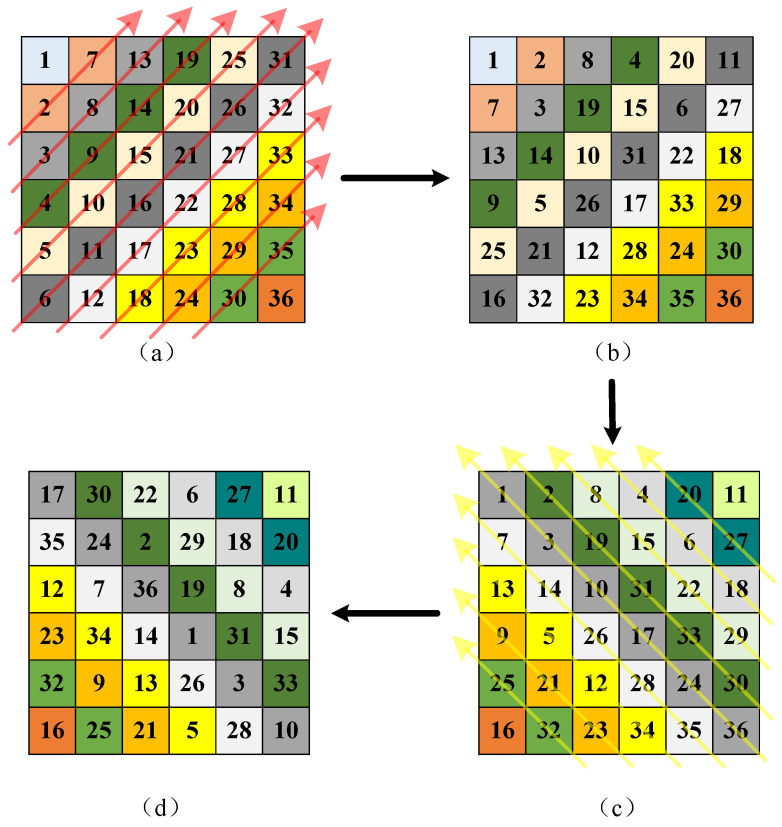
Diagonal cyclic-shift transformation: (**a**) anti-diagonal cyclic shift (direction indicated by red arrows); (**b**) pixels after anti-diagonal cyclic shift; (**c**) main diagonal cyclic shift (direction indicated by yellow arrows); (**d**) pixels after main diagonal cyclic shift.

Figure 5 shows the diagonal cyclic-shift transformation. Taking a 6 × 6 matrix as an example, each anti-diagonal (in the “/” direction) undergoes an independent cyclic shift, with the shift amount controlled by the chaotic sequence X_1_. Subsequently, each main diagonal (in the “\” direction) is independently cyclically shifted, with the shift amount determined by the chaotic sequence X_2_. The orthogonal nature of these two directions enhances the complexity of pixel-position scrambling.

### 2.4. Rotating Dial Model

**Figure 6:**
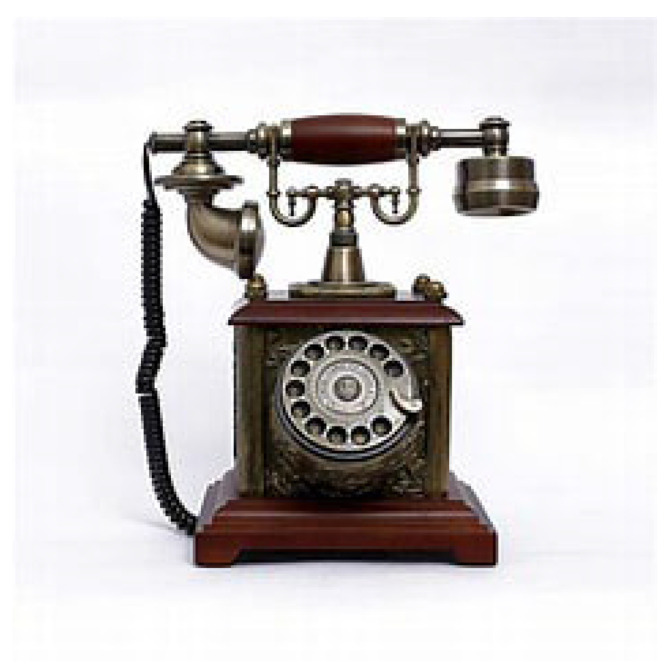
Rotary dial telephone.

Inspired by the rotary dial telephone shown in Figure 6, this paper designs a rotating dial model for the diffusion stage. The dial comprises a superimposed outer ring and inner ring, both divided into 16 equal sectors, with the 16 hexadecimal characters (0–9, A–F) sequentially assigned to each sector. For pixel processing, the index of the high-order hexadecimal character is located on the outer dial. The outer dial characters are rotated clockwise by a chaos-generated offset, and the high-order character is replaced with the new character at the corresponding index. The same operation is applied to the low-order character to complete low-digit substitution. This high- and low-order character replacement is performed for all pixels in the *m* × *n* image.

**Figure 7:**
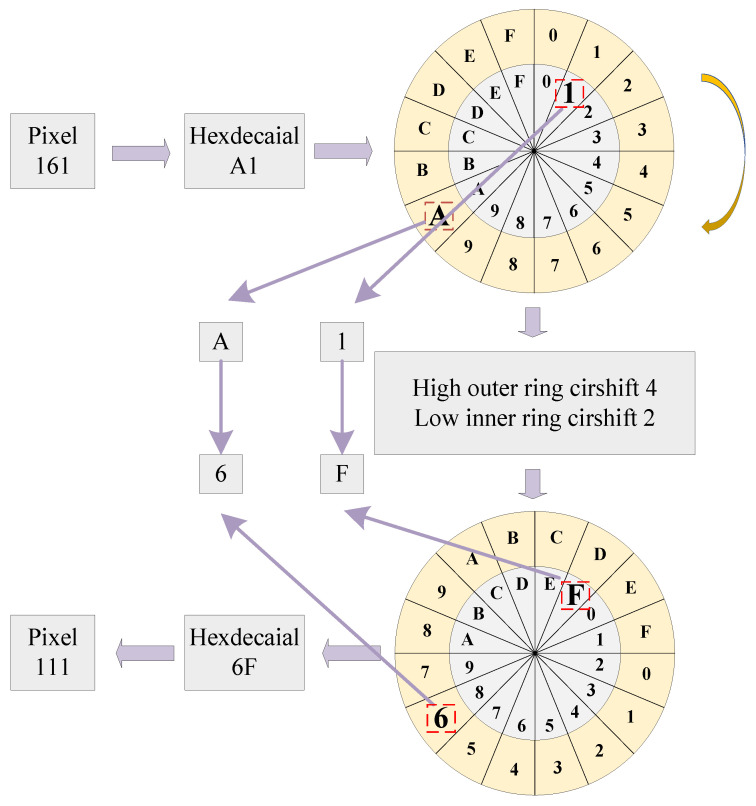
Schematic diagram of the rotating dial model.

Figure 7 illustrates the rotating dial model. Taking pixel 161 as an example, first, the decimal number 161 is converted to the hexadecimal number A1; second, the high-order character A is mapped onto the outer ring dial, and the low-order character 1 is mapped onto the inner ring dial; third, the inner and outer dials are rotated clockwise, with the displacement of each dial controlled by the chaotic system. For example, if the displacement of the inner dial is 2, the inner dial is rotated by 2 units, and the character 1 is replaced with F. If the displacement of the outer dial is 4, the outer dial is rotated by 4 units, and the character A is replaced with 6, yielding the hexadecimal number 6F. Finally, the hexadecimal number 6F is converted to the decimal number 111 to complete the substitution.

## 3. Proposed Image Encryption Algorithm

**Figure 8:**
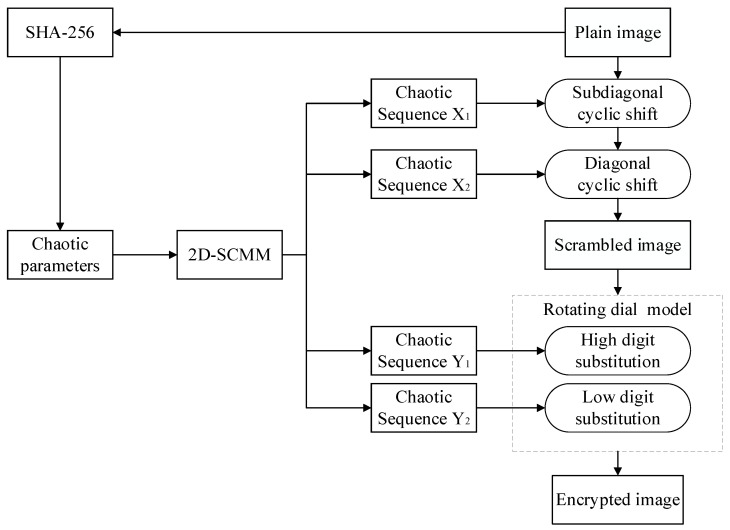
Encryption flowchart.

In this section, an image encryption algorithm using the newly constructed chaotic system is proposed. The encryption algorithm is divided into three phases, namely key generation, scrambling, and diffusion. The flowchart of the image encryption process is shown in Figure 8. The proposed encryption algorithm begins with the extraction of chaotic parameters from the plaintext image using the SHA-256 algorithm. These parameters are then used to perform sub-diagonal and main diagonal scrambling operations on the image. Finally, the scrambled image is processed using a rotating dial model to generate the final encrypted image.

### 3.1. Key Generation

In this stage, the SHA-256 algorithm is first used to generate the hash value of the plaintext image. Algorithm 1 describes the key generation process.
**Algorithm 1** Key generation process**Input:** The plaintext image set *P* and the size *m* × *n* of plaintext image set *P* **Output:** The chaotic sequences *X*_1_, *X*_2_ and *Y*_1_, *Y*_2_ 1: *K*←SHA256(*P*) 2: {*k*_1_, *k*_2_, …, *k*_32_}←*K* 3: {*h*_1_, *h*_2_, …, *h*_32_}←*bin2dec*{*k*_1_, *k*_2_, …, *k*_32_} 4: *μ*←(*h*_1_ + *h*_7_)⊕(*h*_13_ + *h*_19_)⊕(*h*_25_ + *h*_31_)/512 5: *x*_0_←(*h*_2_⊕*h*_8_⊕*h*_14_⊕*h*_20_⊕*h*_26_)/256 6: *y*_0_←(*h*_3_⊕*h*_9_⊕*h*_15_⊕*h*_21_⊕*h*_27_)/256 7: $\mu^{\prime }$←(*h*_4_ + *h*_10_)⊕(*h*_16_ + *h*_22_)⊕(*h*_28_ + *h*_32_)/512 8: ${x_{0}}^{\prime }$←(*h*_5_⊕*h*_11_⊕*h*_17_⊕*h*_23_⊕*h*_29_)/256 9: ${y_{0}}^{\prime }$←(*h*_6_⊕*h*_12_⊕*h*_18_⊕*h*_24_⊕*h*_30_)/256 10: *X*_1_ and *X*_2_←2D-SCMM(*μ*, *x*_0_, *y*_0_) 11: *Y*_1_ and *Y*_2_←2D-SCMM($\mu^{\prime }$, ${x_{0}}^{\prime }$, ${y_{0}}^{\prime }$)

Step 1: Generating hash values

The size of the plaintext image *m* × *n* is obtained. The SHA-256 hash algorithm is employed to compute the hash value of the plaintext image, which is converted into a 256-bit binary sequence. This binary hash sequence is segmented into 32 groups with 8 bits per group, denoted as *H* = (*h*_1_, *h*_2_, …, *h*_32_).

Step 2: Generating keys

Based on the obtained hash value, the initial conditions of the designed 2D-SCMM can be calculated as shown in Equation (8). The arrangement of hash values *h*_i_ in Equation (8) is deliberately designed to enhance the security and effectiveness of key derivation. Each of the six key parameters (*μ*, $\mu^{\prime }$, *x*_0_, ${x_{0}}^{\prime }$, *y*_0_, ${y_{0}}^{\prime }$) is generated by systematically sampling the 32-byte SHA-256 hash output with a fixed stride (e.g., indices 1, 7, 13, 19, 25, 31 for *μ*, spaced at intervals of 6 modulo 32). This stride-based, uniform sampling ensures that all bits of the hash contribute evenly to the keys and helps mitigate potential local correlations within the hash. The combination of addition (+) and XOR (⊕) operations introduces nonlinearity and confusion, strengthening the dependence of the derived keys on the original plaintext (via the hash). Finally, scaling by 512 or 256 normalizes the results to the dynamic ranges required by the chaotic system. This structured approach aims to maximize the utilization of the hash information while ensuring key sensitivity and robustness.

Step 3: Generating chaotic sequences

2D-SCMM iterates 1000 + *m* + *n* times based on the initial values *μ*, *x*_0_, *y*_0_ and discards the first 1000 values of 2D-SCMM. The chaotic sequences *X*_1_ and *X*_2_ are generated with the same length, *m* + *n*. Moreover, 2D-SCMM iterates 1000 + *m* × *n* times based on the initial values $\mu^{\prime }$, ${x_{0}}^{\prime }$, ${y_{0}}^{\prime }$ and discards the first 1000 values of 2D-SCMM. The chaotic sequences *Y*_1_ and *Y*_2_ are generated with the same length, *m* × *n*.

(8) $$\left\{\begin{array}{l} \mu =\frac{\left(h_{1}+h_{7})⊕(h_{13}+h_{19})⊕(h_{25}+h_{31}\right)}{512}\\ x_{0}=\frac{h_{2}⊕h_{8}⊕h_{14}⊕h_{20}⊕h_{26}}{256}\\ y_{0}=\frac{h_{3}⊕h_{9}⊕h_{15}⊕h_{21}⊕h_{27}}{256}\\ \mu^{\prime }=\frac{\left(h_{4}+h_{10})⊕(h_{16}+h_{22})⊕(h_{28}+h_{32}\right)}{512}\\ {x_{0}}^{\prime }=\frac{h_{5}⊕h_{11}⊕h_{17}⊕h_{23}⊕h_{29}}{256}\\ {y_{0}}^{\prime }=\frac{h_{6}⊕h_{12}⊕h_{18}⊕h_{24}⊕h_{30}}{256} \end{array}\right.,$$

### 3.2. Encryption Process

The flowchart of the image encryption process is shown in Figure 8. Algorithm 2 shows the encryption process, and the encryption steps are detailed below.

Step 1: Anti-diagonal cyclic shift

The plaintext image *P* is divided into several one-dimensional sequences along the anti-diagonal direction, denoted as *L*_1_, *L*_2_, …, *L_h_*, where *h* is obtained from Equation (9). After that, the displacement values *steps*_1,_ *steps*_2,_ … *steps_h_*, *j* = 1, 2, …, *h* are calculated for each one-dimensional sequence, where *steps_j_* is given by Equation (10). The permuted image *P*_1_ is obtained.

*h* = *m* + *n* − 1, (9)

*steps_j_* = mod(*floor*((*X*_1_(*j*) + 1) × 10,000), m), *j* = 1, 2, …, *h*, (10)

Step 2: Main diagonal cyclic shift

The image *P*_1_ is divided into several one-dimensional sequences along the main diagonal direction, denoted as ${L^{\prime }}_{1}$, ${L^{\prime }}_{2}$, …, ${L^{\prime }}_{h}$, where *h* is obtained from Equation (9). After that, the displacement values ${{\mathrm{steps}}^{\prime }}_{1}$, ${{\mathrm{steps}}^{\prime }}_{2}$, … ${{\mathrm{steps}}^{\prime }}_{h}$, *j* = 1, 2, …, *h* are calculated for each one-dimensional sequence, where ${{\mathrm{steps}}^{\prime }}_{j}$ is given by Equation (11). The permuted image *P*_2_ is obtained.

*steps′_j_* = mod(*floor*((*X*_2_(*j*) + 1) × 10,000), m), *j* = 1, 2, …, *h*, (11)

Step 3: Integerizing chaotic sequences

Equations (12) and (13) are used to integerize the chaotic sequences *Y*_1_ and *Y*_2_.

*z_i_* = *floor*((*y*_1*i*_ + 1) × 10,000), *i* = 1, 2, …, *mn*, (12)

*w_i_* = *floor*((*y*_2*i*_ + 1) × 10,000), *i* = 1, 2, …, *mn*, (13)

where *y*_1*i*_ ∈ *Y*_1_, *y*_2*i*_ ∈ *Y*_2_. *Z* = {*z_i_*} and *W* = {*w_i_*} are two integer chaotic sequences, both of length *m* × *n*.
**Algorithm 2** Encryption process**Input:** The plaintext image set *P*, the size *m* × *n* of plaintext image set *P* and the chaotic sequences *X*_1_,*X*_2_ and *Y*_1_, *Y*_2_ generated in the Algorithm 1 **Output:** The encrypted image set *E* 1. /*scramble*/ 2. *X*_1_←*floor*((*X*_1_ + 1) × 10,000) 3. *X*_2_←*floor*((*X*_2_ + 1) × 10,000) 4. /* Anti-diagonal cyclic shift */ 5. **for**
*d*←2 **to** 2 × *n*
**do** 6.         **for**
*p*←1 **to** *n*
**do** 7.               *q*←*d* − *p* *8*.         **if**
*q* ≧ 1 **&&** *q* ≦ n 9.                *steps_j_*←*X*_1_; 10.              *P*_1_←*P*(*steps_j_*) 11. /* *P*_1_ refers to the image scrambled by anti-diagonal cyclic shift. */ 12.        **end** 13. **end** 14. /* Main diagonal cyclic shift */ 15. **for**
*d*←−(*n* − 1) **to** (*n* − 1) **do** 16.        **for**
*p*←1 **to** *n*
**do** 17.            *q*←*d* + *p* 18.        **if**
*q* ≧ 1 **&&** *q* ≦ *n* 19.              ${{\mathrm{steps}}^{\prime }}_{j}$←*X*_2_; 20.              *P*_2_←*P*_1_ (${{\mathrm{steps}}^{\prime }}_{j}$) 21. /* *P*_2_ refers to the image scrambled by main diagonal cyclic shift. */ 22.         **end** 23. **end** 24. /*diffusion*/ 25. *Z*←*floor*((*Y*_1_ + 1) × 10,000) 26. *W*←*floor*((*Y*_2_ + 1) × 10,000) 27. *R*←*reshape*(*Z*, *m*, *n*) 28. *S*←*reshape*(*W*, *m*, *n*) 29. *b_jk_*←*dec2hex*(*p_jk_*) 30. **for** *j*←1 **to** *m*
**do** 31.            **for**
*k*←1 **to**
*n*
**do** 32.                *h_jk_*←(*b_jk_*, *R*, *S*) 33.            **end** 34. **end** 35. *e_jk_*←*hex2dec*(*h_jk_*)

Step 4: Chaotic sequence matrixing

*Z* and *W* are converted into two two-dimensional matrices *R* = [*r_jk_*] and *S* = [*s_jk_*], both of size *m* × *n*, in the order of their elements using Equations (14) and (15).

*R* = *reshape*(*Z*, *m*, *n*), (14)

*S* = *reshape*(*W*, *m*, *n*), (15)

where *reshape*(·) is the matrix-dimension reconstruction function.

Step 5: Using the rotating dial model

First, each pixel *p_jk_* of *P*_2_ is converted to the corresponding hexadecimal number *b_jk_* using Equation (16):

*b_jk_* = *dec2hex*(*p_jk_*), *j* = 1, 2, …, *m*, *k* = 1, 2, …, *n*, (16)

where *dec2hex*(·) denotes the decimal-to-hexadecimal conversion function, and the result is an *m* × *n* hexadecimal matrix *B* = [*b_jk_*]. Each element *b_jk_* in matrix *B* is decomposed into a high nibble *c_jk_* and a low nibble *d_jk_*, i.e., *b_jk_* = *c_jk_d_jk_*.

Subsequently, each element in matrix *B* is substituted using the rotating dial model proposed in Section 2.4. Specifically, the high nibble is first right-shifted, followed by a modulo-16 subtraction operation. High-nibble substitution and low-nibble substitution are then performed sequentially on all elements to obtain an *m* × *n* hexadecimal matrix *H* = [*h_jk_*].

Finally, each element *h_jk_* in matrix *H* is converted from hexadecimal into decimal using Equation (17) to generate the final encrypted image *E* = [*e_jk_*]:

*e_jk_* = *hex2dec*(*h_jk_*), *j* = 1, 2, …, *m*, *k* = 1, 2, …, *n*, (17)

where *h_jk_* ∈ *H*, and *hex2dec*(·) denotes the hexadecimal-to-decimal conversion function.

### 3.3. Decryption Process

**Figure 9:**
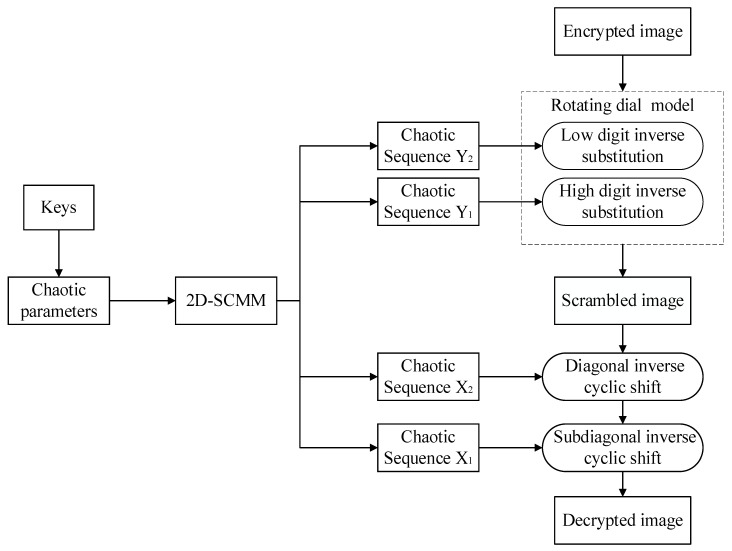
Decryption flowchart.

The flowchart of the decryption process is shown in Figure 9. For the encrypted image *E* of size *m* × *n*, the decryption process is the inverse of the encryption process. The sender sends the initial values *x*_0_, *y*_0_, *x*_0_′, *y*_0_′ and the control parameters *μ*, *μ*′ to the recipient. Algorithm 3 shows the decryption process, and the decryption steps are described below.

Step 1: Generating chaotic sequences

The received keys (*µ*, *x*_0_, *y*_0_) are iterated through 2D-SCMM 1000 + *m* + *n* times and discards the first 1000 values of 2D-SCMM. The chaotic sequences *X*_1_ and *X*_2_ are generated with the same length, *m* + *n*. Similarly, the received keys ($\mu^{\prime }$, ${x_{0}}^{\prime }$, ${y_{0}}^{\prime }$) are iterated through 2D-SCMM 1000 + *m* × *n* times and discards the first 1000 values of 2D-SCMM. The chaotic sequences *Y*_1_ and *Y*_2_ are generated with the same length, *m* × *n*.

Step 2: Inverse rotating dial model

The chaotic matrices *R* and *S* are obtained using Equations (12)–(15), and each element in *E* is then converted into hexadecimal using the *dec2hex* function. Subsequently, each element undergoes a reverse rotating-dial substitution operation according to the values of the chaotic matrices. Finally, each hexadecimal number is converted into decimal using the *hex2dec* function to obtain the image *P_2_*.

Step 3: Main diagonal inverse cyclic shift

The displacement values ${{\mathrm{steps}}^{\prime }}_{j}$, *j* = 1, 2, …, *h* are obtained using Equation (11). A reverse main diagonal cyclic shift is performed on image *P*_2_ to obtain image *P*_1_.

Step 4: Anti-diagonal inverse cyclic shift

The displacement values *steps_j_*, *j* = 1, 2, …, *h* are obtained using Equation (10). Inverse sub-diagonal cyclic shifts are performed on image *P*_1_ to obtain image *P*.
**Algorithm 3** Decryption process**Input:** The encrypted image set *E*, the size *m* × *n* of encrypted image set *E* and the initial values *μ*, *x*_0_, *y*_0_, $\mu^{\prime }$, ${x_{0}}^{\prime }$, ${y_{0}}^{\prime }$ **Output:** the decrypted image set *P* 1. /* Generate the chaotic sequence */ 2. Use *μ*, *x*_0_, *y*_0_, $\mu^{\prime }$, ${x_{0}}^{\prime }$, ${y_{0}}^{\prime }$ to generate chaotic sequence *X*_1_, *X*_2_ and *Y*_1_, *Y*_2_ 3. /* inverse diffusion */ 4. *Z*←*floor*((*Y*_1_ + 1) × 10,000) 5. *W*←*floor*((*Y*_2_ + 1) × 10,000) 6. *R*←*reshape*(*Z*, *m*, *n*) 7. *S*←*reshape*(*W*, *m*, *n*) 8. *h_jk_*←*dec2hex*(*e_jk_*) 9. **for**
*j*←1 **to**
*m*
**do** 10.        **for**
*k*←1 **to**
*n*
**do** 11.              *b_jk_*←(*h_jk_*,−*R*,−*S*) 12.        **end** 13. **end** 14. *p_jk_*←*hex2dec*(*b_jk_*) 15. /* inverse scramble */ 16. *X*_1_←*floor*((*X*_1_ + 1) × 10,000) 17. *X*_2_←*floor*((*X*_2_ + 1) × 10,000) 18. **for** d←−(*n* − 1) **to** (*n* − 1) **do** 19.        **for**
*p*←1 **to**
*n*
**do** 20.              *q*←*d* + *p* 21.        **if**
*q* ≧ 1 **&&**
*q* ≦ n 22.               ${{\mathrm{steps}}^{\prime }}_{j}$←*X*_2_; 23.               *P*_1_←*P*_2_ (−${{\mathrm{steps}}^{\prime }}_{j}$) 24.        **end** 25. **end** 26. **for**
*d*←2 **to** 2 × *n*
**do** 27.        **for**
*p*←1 **to**
*n*
**do** 28.             *q*←*d* − *p* 29.        **if**
*q* ≧ 1 **&&**
*q* ≦ n 30.               *steps_j_*←*X*_1_; 31.               *P*←*P*_1_ (−*steps_j_*) 32.        **end** 33. **end**

## 4. Experimental Results

**Figure 10:**
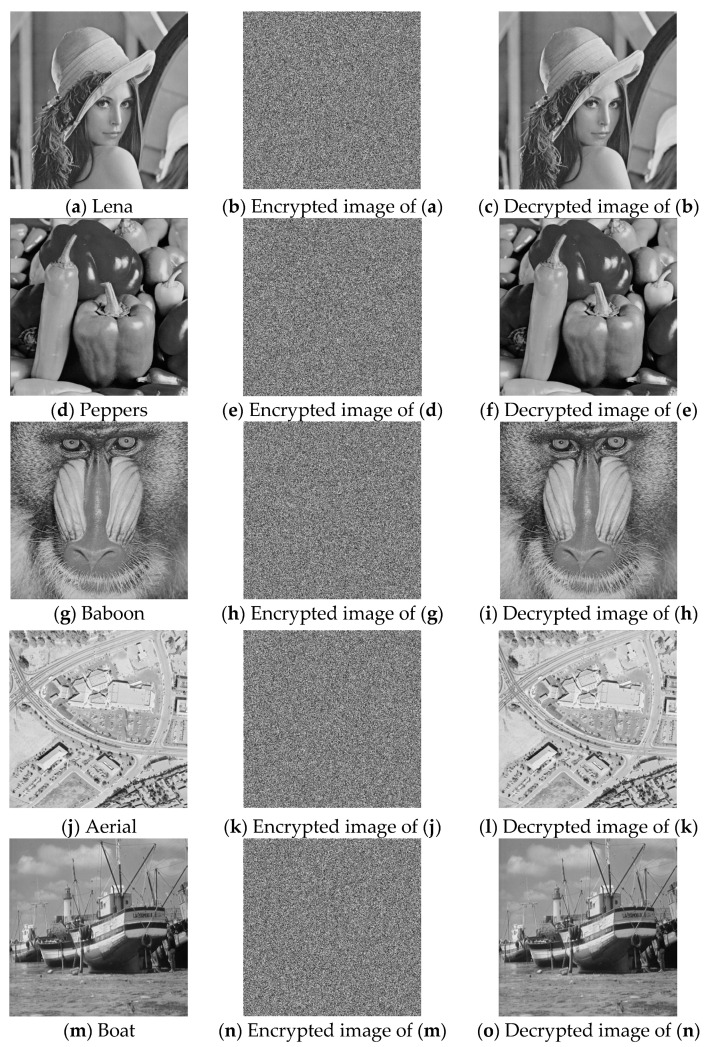
Experimental results.

To evaluate the performance of the proposed encryption algorithm, MATLAB R2023b was used as the simulation platform. The experimental configuration was as follows: AMD Ryzen 7 7840H with Radeon 780M Graphics, 3.80 GHz, and 16 GB of RAM. The images used in this paper were obtained from the USC Signal and Image Processing Institute image database (http://sipi.usc.edu/database, accessed on 12 March 2026). Figure 10 shows the results of encrypting and decrypting 512 × 512 images of Lena, Peppers, Baboon, Aerial, and Boat using the proposed algorithm. It can be seen that the encrypted images are cluttered and noise-like, effectively concealing the plaintext information. Meanwhile, the decrypted images are identical to the plaintext images, which shows that the proposed algorithm can recover the plaintext information without distortion. Therefore, information security is effectively ensured.

### 4.1. Key Space Analysis

The key space refers to the total number of all possible keys. Generally, a key space is considered secure if its scale exceeds 2^100^. A larger key space indicates stronger resistance of the algorithm to brute-force attacks.

(1) From the perspective of key generation, the key is a 256-bit hash digest generated by the SHA-256 algorithm; thus, the key space of the encryption algorithm is 2^256^.
(2) From the perspective of chaotic parameters, the key adopted in the proposed algorithm consists of the control parameters *μ*, $\mu^{\prime },$ and the initial values *x*_0_, *y*_0_, ${x_{0}}^{\prime }$, and ${y_{0}}^{\prime }$ of the chaotic system. Given that the computational precision of the computer is 10^−14^, the key space of the encryption algorithm is calculated as 10^14 × 6^ ≈ 2^279^ >> 2^100^.

Therefore, the final key space is determined as the minimum value of the two aforementioned key spaces, namely 2^256^. Since 2^256^ >> 2^100^, the proposed encryption algorithm possesses excellent resistance to brute-force attacks.

### 4.2. Key Sensitivity Analysis

**Figure 11:**
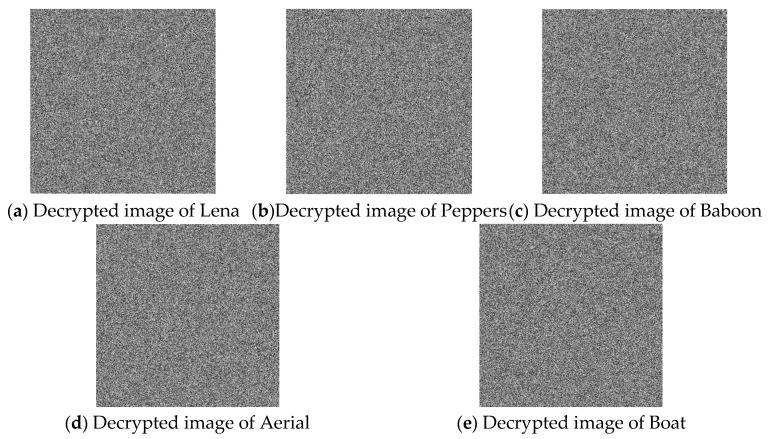
Images decrypted using incorrect keys.

Key sensitivity refers to the property that the encrypted image changes drastically when there is an extremely small change in the key, or that the decrypted image becomes irrelevant to the original image, making it impossible to recover the correct plaintext image. In this algorithm, the key consists of the control parameter *μ*, $\mu^{\prime }$, and the initial conditions *x*_0_, *y*_0_, ${x_{0}}^{\prime }$, and ${y_{0}}^{\prime }$. After encrypting the plaintext image using these keys, the ciphertext image is decrypted using an incorrect key. In order to better evaluate key sensitivity, decryption is performed by perturbing *μ* by a very small value of 10^−14^. Figure 11 shows the decrypted image using the incorrect key, and it can be seen that the decrypted image is completely inconsistent with the original image.

### 4.3. Histogram Analysis

**Figure 12:**
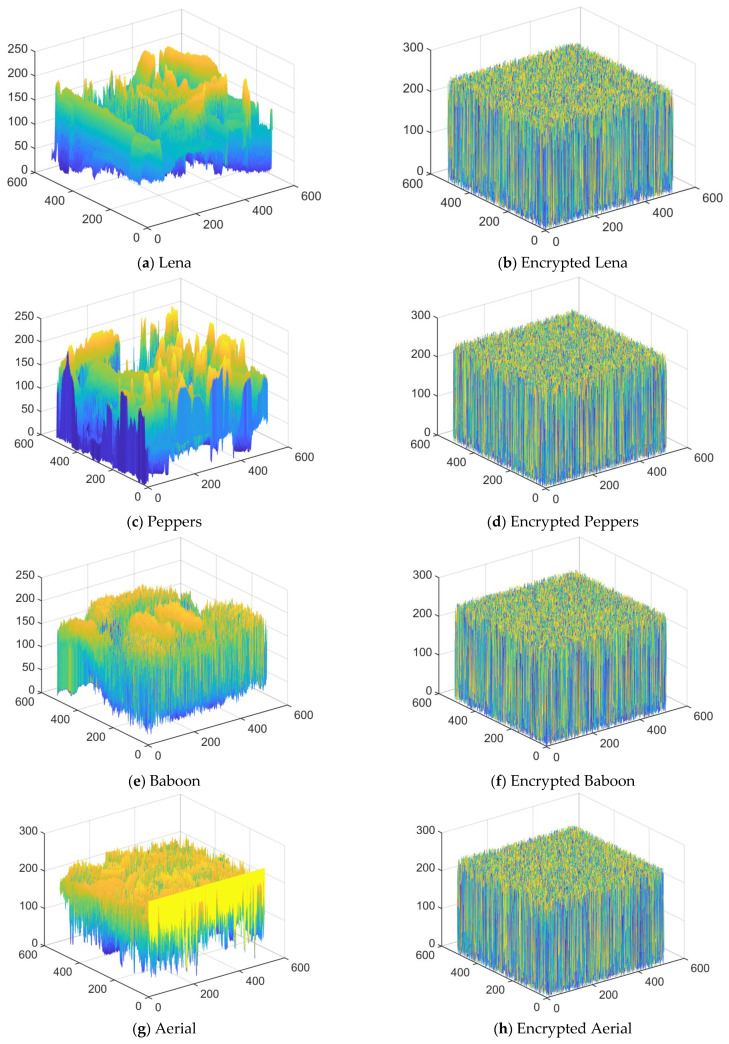
Histograms of plaintext images and encrypted images.

A histogram is a visual depiction of the pixel-value distribution in an image, i.e., it shows the frequency of each gray level in the image. If all gray-value frequencies in a ciphertext image are approximately equal, then its corresponding histogram should be flat and resistant to statistical analysis attacks. The histograms of the plaintext and ciphertext images are shown in Figure 12. It can be seen that the histogram of the plaintext image fluctuates significantly, which makes it easy for an attacker to perform statistical analysis attacks. However, the histogram of the ciphertext image is relatively flat, indicating that the ciphertext image effectively conceals the information in the plaintext image. Therefore, the proposed encryption algorithm can effectively resist statistical analysis attacks.

### 4.4. Information Entropy

Information entropy is an important measure of the distribution of pixel grayscale values in an encrypted image. An image with higher information entropy indicates a more uniform distribution of pixel values, whereas an image with lower information entropy indicates a more concentrated distribution of pixel values. Information entropy is calculated as follows:

(18) $$H(X)=\sum_{i=1}^{n}p(x_{i})\log_{2}\frac{1}{p(x_{i})},$$

where *n* represents the number of pixels, *x_i_* ∈ [0, 255] represents the pixel value of the grayscale image, and *p*(*x_i_*) represents the frequency of occurrence of pixel *x_i_*. Generally speaking, the theoretical maximum value of information entropy is 8. The standard deviation of different images is calculated as shown in Table 2. The information entropy values of the proposed algorithm and similar existing image encryption algorithms are shown in Table 3. The information entropy of the proposed algorithm is close to the theoretical value; therefore, the pixel distribution of the encrypted image is highly uniform with high randomness, and it can better resist information entropy attacks.

### 4.5. Correlation Analysis

Correlation analysis quantifies the degree of association between two or more variables and their direction by calculating the correlation coefficient (e.g., Pearson’s coefficient). It is widely used in data research and image processing, for example, to assess the continuity or anomalous features of neighboring pixels. The correlation coefficient is calculated as follows:

(19) $$r_{ab}=\frac{\sum_{i=1}^{n}\left(a_{i}-\bar{a})(b_{i}-\bar{b}\right)}{\sqrt{\sum_{i=1}^{n}{\left(a_{i}-\bar{a}\right)}^{2}}\sqrt{\sum_{i=1}^{n}{\left(b_{i}-\bar{b}\right)}^{2}}}$$

where *r_ab_* is the correlation coefficient and $\bar{a\;}$ and $\bar{b}$ are the means.

**Figure 13:**
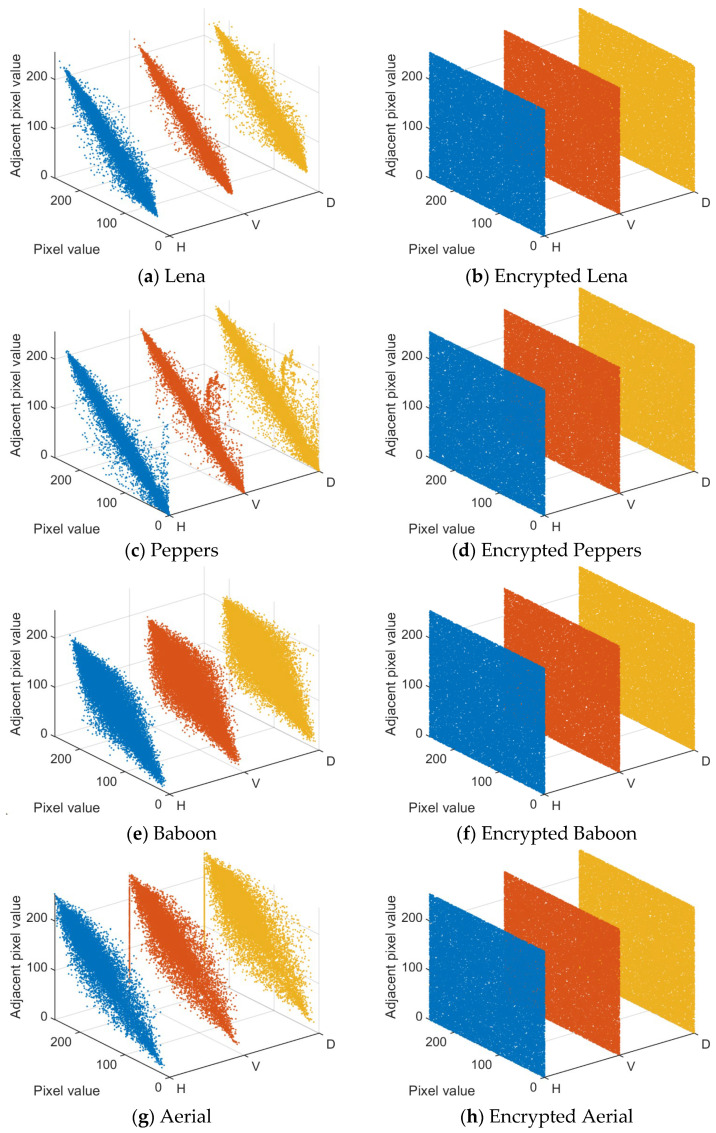
Correlation analysis of plaintext images and encrypted images in three directions.

Figure 13 illustrates the correlation of neighboring pixels in the horizontal, vertical, and diagonal directions of the image before and after encryption. In the 3D coordinate system, the *x*-axis marks the spatial direction of the pixel (H horizontal, V vertical, D diagonal), the *y*-axis represents the gray value of the target pixel, and the *z*-axis corresponds to the gray value of its neighboring pixels. For plaintext images, due to the highly similar gray values of neighboring pixels, the data points are centrally distributed near a linear trajectory, regardless of the horizontal, vertical, or diagonal directions, reflecting a strong spatial correlation. In the encrypted image, on the other hand, the inter-pixel correlation is significantly disrupted, and the data points show an irregular discrete distribution. The more scattered the distribution (i.e., the higher the dispersion of the correlation map), the stronger the degree of pixel misalignment, and the more significant the effect of the encryption algorithm on perturbing image features. Table 4 shows the correlation coefficients of plaintext and encrypted images. The correlation coefficients of plaintext images are close to 1, whereas the correlation coefficients of encrypted images are close to 0. Therefore, the proposed algorithm is effective in removing the correlation between image pixels.

### 4.6. Differential Attacks

**Figure 14:**
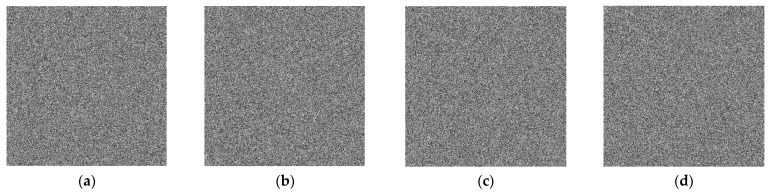
Difference images: (**a**) Lena; (**b**) Peppers; (**c**) Baboon; (**d**) Aerial.

A secure encryption algorithm should be highly sensitive to small changes in pixel values in the plaintext image, i.e., the ciphertext image obtained after changing any pixel value in the plaintext image and the ciphertext image obtained from the original plaintext image should be completely different. The Number of Pixels Change Rate (NPCR) and the Unified Average Changing Intensity (UACI) are two important indicators used to evaluate the sensitivity of encryption algorithms to plaintext, i.e., their ability to resist differential attacks. The ideal values of NPCR and UACI are about 99.6094% and 33.4635%, respectively. The closer the NPCR and UACI values are to the ideal values, the more sensitive the encryption algorithm is to the plaintext image, i.e., the more resistant the encryption algorithm is to differential attacks. NPCR and UACI can be computed as follows:

(20) $$NPCR=\frac{\sum_{i,j}D(i,j)}{M\times N}\times 100\%\;,$$

(21) $$UACI=\frac{1}{M\times N}\left[\sum_{i,j}\frac{\left|c_{1}(i,j)-c_{2}(i,j)\right|}{255}\right]\times 100\%\;,$$

where *C*_1_ and *C*_2_ represent the encrypted image of the plaintext image and the encrypted image of the plaintext image after a pixel value has been changed, respectively. During the experiments, a pixel value is randomly selected in the plaintext image and modified. The difference image is generated by calculating the absolute difference between the two cipher images obtained by altering a single pixel in the plaintext image. Figure 14 shows the difference images. It exhibits obvious and uniform differences, from which no meaningful information can be identified. Table 5 shows the test results of the two encrypted images obtained before and after the pixel value change using the proposed encryption algorithm.

### 4.7. Chosen Plaintext Attacks

**Figure 15:**
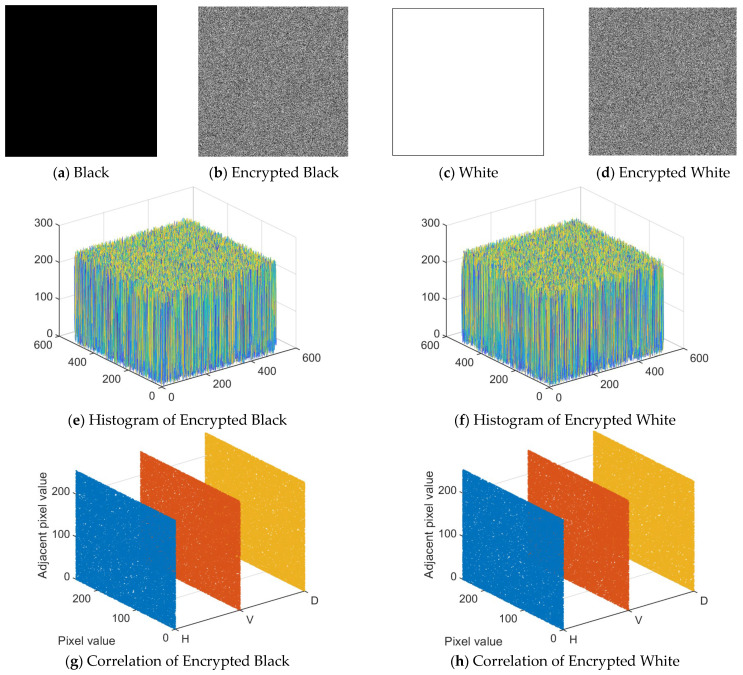
Encrypted images, histograms, and correlation analysis for black and white images.

Plaintext attacks are also among the methods used by attackers to crack ciphertext images. The effectiveness of a chosen plaintext attack is usually evaluated by encrypting a special image that is either all black or all white. Figure 15 shows the encrypted images, histograms, and correlation analysis of the all-black and all-white images, and the resulting encrypted images are cluttered and noisy, containing no meaningful information, which indicates that the proposed algorithm has strong resistance to plaintext attacks.

### 4.8. Cropping Attacks

**Figure 16:**
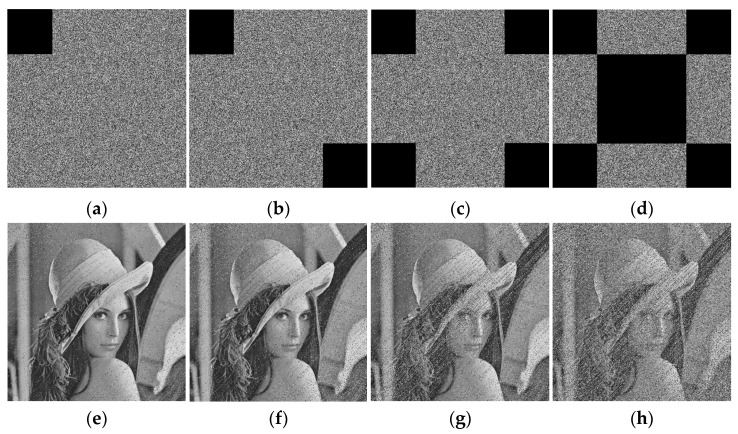
Cropping attack analyses of Lena: (**a**) crop 1/16; (**b**) crop 1/8; (**c**) crop 1/4; (**d**) crop 1/2; (**e**) decrypted image of (**a**); (**f**) decrypted image of (**b**); (**g**) decrypted image of (**c**); (**h**) decrypted image of (**d**).

**Figure 17:**
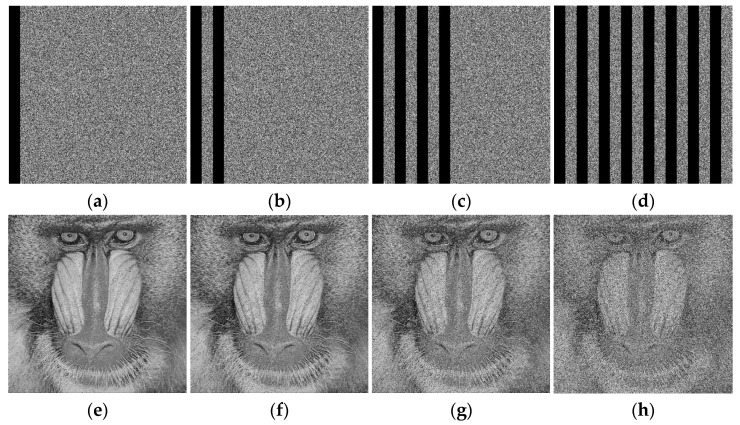
Cropping attack analyses of Baboon: (**a**) crop 1/16; (**b**) crop 1/8; (**c**) crop 1/4; (**d**) crop 1/2; (**e**) decrypted image of (**a**); (**f**) decrypted image of (**b**); (**g**) decrypted image of (**c**); (**h**) decrypted image of (**d**).

**Figure 18:**
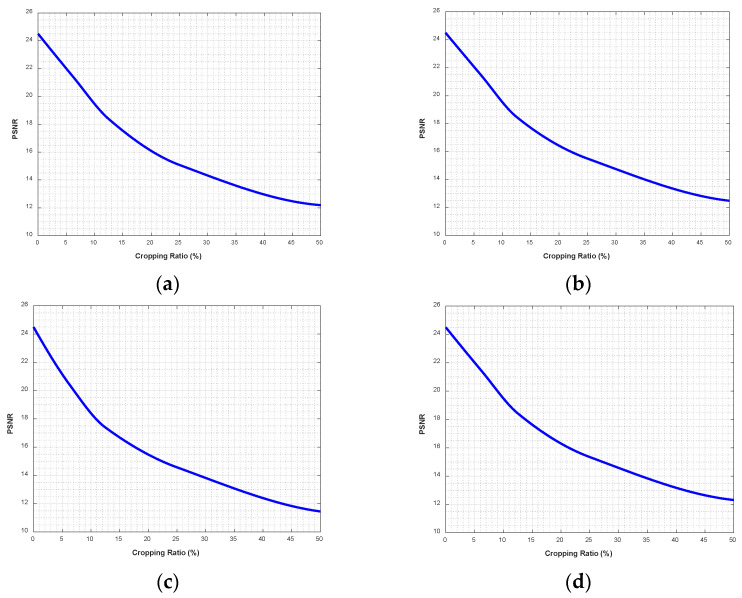
PSNR performance degradation curves under cropping ratios: (**a**) Baboon; (**b**) Boat; (**c**) Peppers; (**d**) Aerial.

During the transmission of encrypted images, the images are susceptible to channel interference, which changes the pixel values of the images. In this experiment, the missing data is filled with zeros. Decryption is performed on the cropped encrypted image. The decryption results for Lena and the Baboon are shown in Figure 16 and Figure 17, respectively. Also, for the other images cropped according to Lena’s cropping format, the corresponding PSNR values are shown in Table 6. The PSNR performance degradation curve with cropping ratio is shown in Figure 18. It can be seen that the decrypted images still retain the information of the original images. Therefore, it can be concluded that the proposed algorithm has good resistance to cropping attacks.

### 4.9. Noise Attacks

**Figure 19:**
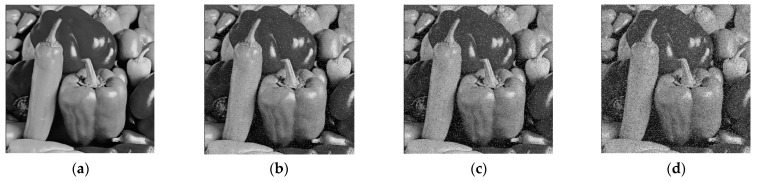
Noise attack results: (**a**) decrypted Baboon without noise; (**b**) decrypted Baboon with 0.05 noise density; (**c**) decrypted Baboon with 0.1 noise density; (**d**) decrypted Baboon with 0.2 noise density.

**Figure 20:**
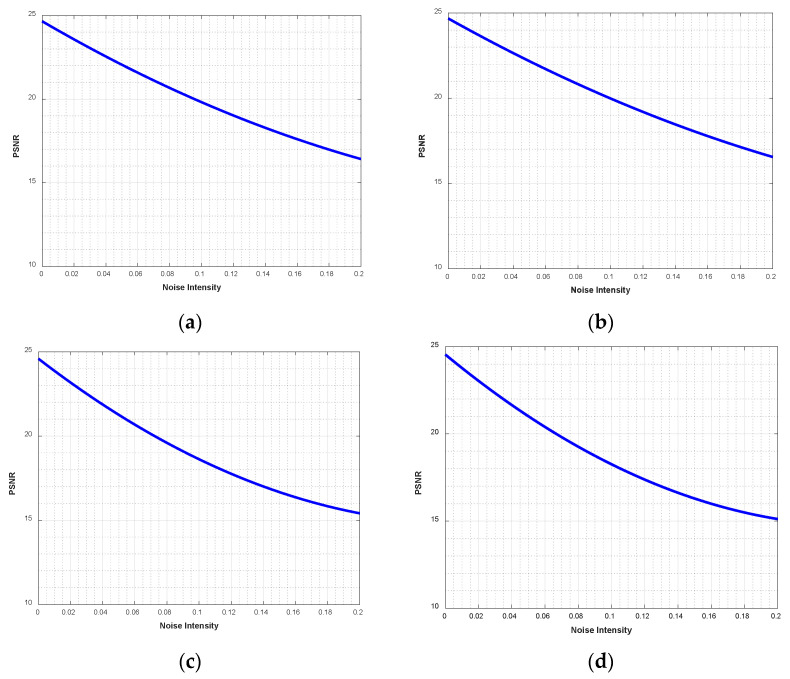
PSNR performance degradation curves under noise intensity: (**a**) Baboon; (**b**) Boat; (**c**) Peppers; (**d**) Aerial.

The ciphertext image is easily affected by noise during transmission, which may affect the correct decryption of the image. Taking the Peppers grayscale image of size 512 × 512 as an example, Figure 19b–d show the decrypted images with different densities of salt-and-pepper noise, where the noise densities are 0.05, 0.1, and 0.2, respectively. Meanwhile, different densities of salt-and-pepper noise are also added to the other images, and the corresponding PSNR values are shown in Table 7. The PSNR performance degradation curves under noise intensity are shown in Figure 20. It can be seen that the decrypted images remain recognizable, which indicates that the proposed encryption algorithm can resist a certain degree of salt-and-pepper noise attack.

### 4.10. Computational Complexity Analysis

**Figure 21:**
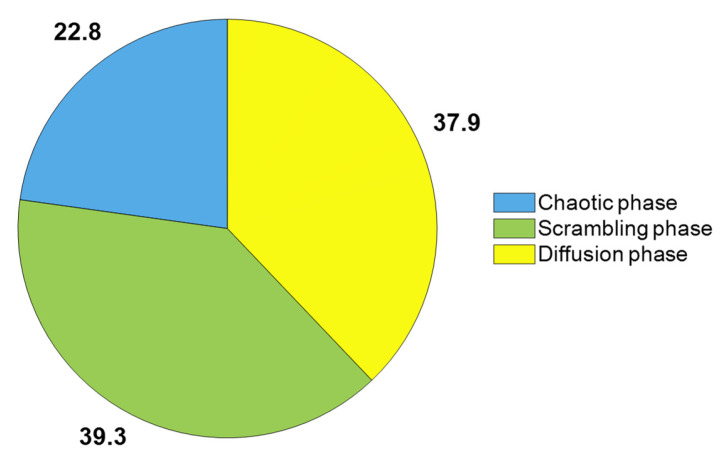
Time consumed for each process of the proposed algorithm (%).

Computational complexity measures the computational resources required by an algorithm. The proposed encryption algorithm consists of three main components: a chaotic phase, a scrambling phase, and a diffusion phase. Assuming the size of the encrypted image is *m* × *n*, the complexity of the chaotic phase is *O*(*m* × *n* + *m* + *n*), and since *m* + *n* ≪ *m* × *n*, the complexity of this stage remains *O*(*m* × *n*). For the scrambling phase, the complexity is *O*(*m* × *n*). For the diffusion phase, the complexity is *O*(*m* × *n*). Therefore, the overall computational complexity of the proposed encryption algorithm is *O*(3 × *m* × *n*). Figure 21 shows the execution times of specific encryption processes as a percentage of the total encryption time.

## 5. Conclusions and Outlook

In this paper, a novel two-dimensional chaotic system (2D-SCMM) is proposed and applied to image encryption, exhibiting excellent randomness, high complexity, and strong initial condition sensitivity through comprehensive experiments (including NIST tests and statistical analysis), thereby meeting the core requirements for image encryption. The rotating dial model is innovatively introduced in the diffusion phase to enhance sensitivity and anti-attack capability, while a cyclic-shift diagonal-transform scrambling method is designed to reduce pixel correlation. The integration of these components provides a new design approach for secure image encryption. Experimental results confirm the algorithm’s excellent information-hiding capability and resistance to common attacks (e.g., noise, cropping), with high security and practicality.

Future work will include chaos game representation [50] (CGR) analysis to verify the ergodicity and structural complexity of the 2D chaotic map, hybridizing continuous chaotic dynamics with discrete mechanisms for robustness [51] and parallel implementation, extending the scheme to color image encryption with inter-channel coupling, and optimizing computational efficiency without compromising core security principles. Moreover, we will implement the algorithm on GPU or FPGA platforms to evaluate its running speed, resource consumption, and energy consumption, thereby further verifying its practicability in real-time image transmission scenarios.

## Figures and Tables

**Table 1:** NIST test results of the 2D-SCMM.

Statistical Test	*p*-Value		Result
	*x*(*n*)	*y*(*n*)	
Frequency	0.7278	0.1014	Passed
Block Frequency	0.4370	0.4329	Passed
Runs	0.0982	0.8582	Passed
Longest Run	0.7369	0.8364	Passed
Rank	0.0235	0.0192	Passed
FFT	0.0255	0.3840	Passed
Non-overlapping Template	0.1347	0.6563	Passed
Overlapping Template	0.8452	0.8015	Passed
Universal	0.6913	0.0992	Passed
Linear Complexity	0.6032	0.3675	Passed
Serial Test (*p*-value 1)	0.0298	0.0770	Passed
Serial Test (*p*-value 2)	0.0820	0.2228	Passed
Approximate Entropy	0.3201	0.4215	Passed
Cumulative Sums (forward)	0.9998	0.9963	Passed
Cumulative Sums (reverse)	0.9751	0.9925	Passed
Excursions (X = 1)	0.1938	0.8878	Passed
Excursions Variant (X = 1)	0.0366	0.3228	Passed

**Table 6:** PSNR values under cropping attacks.

Cropping Area	Lena	Baboon	Peppers	Boat
1/16	21.3872	21.4738	20.4391	21.4060
1/8	18.3801	18.4851	17.4011	18.4265
1/4	15.0848	15.5026	14.5755	15.3446
1/2	12.1954	12.4849	11.4521	12.3138

**Table 7:** PSNR values under noise attacks.

Noise Intensity	Baboon	Boat	Peppers	Aerial
0.05	22.4603	22.6652	21.4767	21.1409
0.1	19.5104	19.6235	18.4525	18.1560
0.2	16.4660	16.6153	15.4368	15.1309

**Table 2:** Standard deviation of different images.

Algorithm	Image	Plain	Cipher
Proposed	Lena	47.8520	73.7475
	Peppers	59.4030	73.7029
	Baboon	42.3010	73.6333
	Aerial	39.4444	73.7257
	Boat	46.6772	73.6771
	Tank	27.0568	73.6529
	Clock	57.2492	73.8381
	Airplane	22.1186	73.7225
	Truck	27.0634	73.5983

**Table 3:** Entropy of different images.

Algorithm	Image	Plain	Cipher
Proposed	Lena	7.4456	7.9993
	Peppers	7.5715	7.9993
	Baboon	7.3579	7.9992
	Aerial	6.9940	7.9993
	Boat	7.1914	7.9993
	Tank	5.4957	7.9992
	Clock	6.7057	7.9993
	Airplane	4.0045	7.9991
	Truck	6.0274	7.9993
Ref. [32]	Lena	7.4456	7.9967
	Peppers	7.5715	7.9994
Ref. [34]	Baboon	7.3579	7.9974
	Peppers	7.5715	7.9977
Ref. [45]	Peppers	7.5715	7.9992
	Baboon	7.3579	7.9993
	Boat	7.1914	7.9991
Ref. [46]	Peppers	7.5715	7.9993
	Baboon	7.3579	7.9994
	Boat	7.1914	7.9992
Ref. [47]	Peppers	7.5715	7.9975
Ref. [48]	Peppers	7.5715	7.9948
Ref. [49]	Peppers	7.5715	7.9971

**Table 4:** Correlation coefficients of different images.

Plain Image	Test Image	Horizontal	Vertical	Diagonal
Lena	Plain image	0.9719	0.9849	0.9591
	Encrypted image	0.0007	0.0021	0.0044
Peppers	Plain image	0.9760	0.9809	0.9663
	Encrypted image	0.0025	0.0024	−0.0037
Baboon	Plain image	0.8667	0.7498	0.7158
	Encrypted image	0.0005	0.0044	−0.0009
Aerial	Plain image	0.9007	0.8534	0.7889
	Encrypted image	−0.0027	0.0016	0.0018
Boat	Plain image	0.9383	0.9715	0.9224
	Encrypted image	0.0013	−0.0024	0.00049
Peppers [19]	Encrypted image	0.0027	−0.0013	−0.0014
Baboon [19]	Encrypted image	0.0034	0.0022	0.0057
Lena [31]	Encrypted image	0.0027	−0.0023	−0.0018
Peppers [31]	Encrypted image	−0.0013	0.0016	0.0013
Baboon [34]	Encrypted image	−0.0051	−0.0082	0.0060
Peppers [45]	Encrypted image	0.0024	−0.0011	−0.0002
Baboon [45]	Encrypted image	−0.0025	−0.0026	−0.0031
Boat [45]	Encrypted image	−0.0024	−0.0050	0.0084
Peppers [48]	Encrypted image	0.0026	−0.0037	0.0017

**Table 5:** NPCR and UACI results.

Test Image	NPCR	UACI
Lena	99.6281	33.4608
Peppers	99.6059	33.4366
Baboon	99.5922	33.4687
Aerial	99.5846	33.3913
Boat	99.5941	33.4169

## Data Availability

Some or all of the data generated or used in this study are available from the corresponding author upon reasonable request.
